# Carbon Nanostructured Immunosensing of Anti-SARS-CoV-2 S-Protein Antibodies

**DOI:** 10.3390/molecules28248022

**Published:** 2023-12-09

**Authors:** Jarid du Plooy, Branham Kock, Nazeem Jahed, Emmanuel Iwuoha, Keagan Pokpas

**Affiliations:** SensorLab, Department of Chemistry, University of the Western Cape, Robert Sobukwe Road, Bellville, Cape Town 7535, South Africa

**Keywords:** SARS-CoV-2, carbon nanomaterials, electrochemical immunosensor, point-of-care device, label-free detection

## Abstract

The rampant spread and death rate of the recent coronavirus pandemic related to the SARS-CoV-2 respiratory virus have underscored the critical need for affordable, portable virus diagnostics, particularly in resource-limited settings. Moreover, efficient and timely monitoring of vaccine efficacy is needed to prevent future widespread infections. Electrochemical immunosensing poses an effective alternative to conventional molecular spectroscopic approaches, offering rapid, cost-effective, sensitive, and portable electroanalysis of disease biomarkers and antibodies; however, efforts to improve binding efficiency and sensitivity are still being investigated. Graphene quantum dots (GQDs) in particular have shown promise in improving device sensitivity. This study reports the development of a GQD-functionalized point-of-contamination device leveraging the selective interactions between SARS-CoV-2-specific Spike (S) Protein receptor binding domain (RBD) antigens and IgG anti-SARS-CoV-2-specific S-protein antibodies at screen-printed carbon electrode (SPCE) surfaces. The immunocomplexes formed at the GQD surfaces result in the interruption of the redox reactions that take place in the presence of a redox probe, decreasing the current response. Increased active surface area, conductivity, and binding via EDC/NHS chemistry were achieved due to the nanomaterial inclusion, with 5 nm, blue luminescent GQDs offering the best results. GQD concentration, EDC/NHS ratio, and RBD S-protein incubation time and concentration were optimized for the biosensor, and inter- and intra-screen-printed carbon electrode detection was investigated by calibration studies on multiple and single electrodes. The single electrode used for the entire calibration provided the best results. The label-free immunosensor was able to selectively detect anti-SARS-CoV-2 IgG antibodies between 0.5 and 100 ng/mL in the presence of IgM and other coronavirus antibodies with an excellent regression of 0.9599. A LOD of 2.028 ng/mL was found, offering comparable findings to the literature-reported values. The detection sensitivity of the sensor is further compared to non-specific IgM antibodies. The developed GQD immunosensor was compared to other low-oxygen content carbon nanomaterials, namely (i) carbon quantum dot (CQD), (ii) electrochemically reduced graphene oxide, and (iii) carbon black-functionalized devices. The findings suggest that improved electron transfer kinetics and increased active surface area of the CNs, along with surface oxygen content, aid in the detection of anti-SARS-CoV-2 IgG antibodies. The novel immunosensor suggests a possible application toward monitoring of IgG antibody production in SARS-CoV-2-vaccinated patients to study immune responses, vaccine efficacy, and lifetime to meet the demands for POC analysis in resource-limited settings.

## 1. Introduction

The COVID-19 pandemic, part of the coronavirus family stemming from the highly contagious severe acute respiratory syndrome coronavirus 2 (SARS-CoV-2), has had profound impacts on both human health and socioeconomic activity globally. It has accounted for millions of deaths to date. The virus’s rapid mutation has given rise to numerous variants such as B.1.1.7 (Alpha), B.1.351 (Beta), B.1.617.2 (Delta), P.1 (Gamma), and B.1.1.529 (Omicron) [1]. Common symptoms of infection encompass fever, dry cough, and fatigue, while less frequent but serious manifestations include aches, sore throat, diarrhea, conjunctivitis, headaches, and loss of taste and smell [2]. Given its extensive transmission, health complications, and elevated mortality rate, it is imperative to establish robust healthcare systems and deploy advanced devices for effective monitoring of SARS-CoV-2 spread.

To better combat the virus, technological advancements in healthcare have played a pivotal role. The foremost testing methods for COVID-19 are reverse transcription-polymerase chain reaction (RT-PCR) and the Rapid Antigen Detection Test (RADT). These techniques can adeptly detect SARS-CoV-2 RNA and specific S and N proteins, respectively, with good sensitivity and reliability. Additionally, researchers are exploring other approaches such as enzyme-linked immunosorbent assays (ELISAs) and lateral flow immunoassays (LFIAs) for COVID-19 detection [3]. However, given their often-extended processing times, need for expensive equipment, labor-intensive sample preparation, and demand for skilled personnel, there is a pressing need for alternatives to molecular spectroscopic testing, especially in resource-constrained settings. Electrochemical biosensors provide a highly selective approach for the detection of SARS-CoV-2 biomarkers by incorporating specialized bioreceptors to monitor binding efficiency. Coupling electrochemistry with biosensing allows for a cost-effective detection strategy with little instrument requirements and is commonly used over colorimetric, spectroscopic, and other techniques. This has led to a surge of interest in electrochemical immunoassays as a viable alternative bioanalytical technique over their aptamer, enzyme, RNA, and other bioreceptor counterparts. These assays rely on antigen–antibody interactions, where a current response is observed, enabling low-cost and portable detection with excellent selectivity [4,5,6].

Label-free immunoassays, in comparison to their labeled detection counterparts, operate by measuring redox changes resulting from indirect biomolecule binding. They offer significant advantages due to their speed, cost-effectiveness, simplicity, and minimal pre-reaction requirements [7]. Typically, redox probes are used as an indirect measure of biomarker detection with ferri/ferrocyanide being the most employed. As a result, they have found wide application in antigen monitoring with numerous works reporting their use at different substrates [8,9,10,11,12,13,14]. Antibody testing has also gained popularity in the early detection of coronavirus and assessing vaccine effectiveness. Various methods, ranging from simple to complex systems, have been proposed for electrochemical immunosensing of anti-SARS-CoV-2 S and N protein antibodies, with different functionalities reporting different results. Notably, promising approaches like electrochemical and optical techniques have been employed in designing miniaturized immunosensors, which are fast, accurate, and user-friendly point-of-care (POC) devices for antibody detection [15]. For instance, Braz et al. introduced a selective detection method for anti-S-protein using a Au-binding peptide (Pept/Au) as the biorecognition element, achieving detection in the ng·mL^−1^ range on screen printed carbon electrodes [16]. Timilsina et al. utilized a nanocomposite of antifouling pentaamine-modified graphene nanoflakes (prGOx) cross-linked with glutaraldehyde (GA) for the detection of anti-human-SARS-CoV-2 IgG-HRP. This approach not only increased conductivity but also limited nonspecific binding [17]. In another study by Peng et al., a sandwich assay configuration employing a biotinylated RBD protein as the capture probe was used to monitor ALP-conjugated detection of IgG and IgM antibodies in human serum using chronoamperometry. This method allowed for detection in a wide range, from ng·mL^−1^ to µg·mL^−1^, with long-term stability [18]. Masterson et al. presented a label-free nanoplasmonic biosensor-based multiplex quantitative detection test for 10 different SARS-CoV-2 biomarkers within one biosensing platform. Their novel nanoplasmonic biosensor demonstrated high specificity, effectively minimizing false responses [19]. Najja et al. designed a 3D-printed microfluidic chip for lab-on-a-chip saliva detection of SARS-CoV-2 RNA and antibodies. This approach enabled accurate detection within 2 h of input [20]. Rashed et al. demonstrated the detection of anti-SARS-CoV-2 monoclonal antibody CR3022 in the µg.mL^−1^ range using a 16-well plating with sensing electrodes functionalized with RBD protein [21]. Finally, Yakoh et al. introduced a novel label-free electrochemical immunosensor on paper, the only one of its kind for detecting SARS-CoV-2 antibodies. This method immobilized the SARS-CoV-2 Spike-protein (S-protein) onto graphene oxide, with the detection principle relying on the immune complex formed between the SARS-CoV-2 antibody and antigen, which interrupts the redox conversion of Fe(CN)_6_^3−/4−^, resulting in a decrease in current response. Graphene oxide played a crucial role in improving bioreceptor binding through its oxygen-containing moieties and demonstrates one of the few studies for antibody detection relying on carbon nanostructures [22]. These advancements collectively represent a diverse and innovative approach to SARS-CoV-2 antibody immunosensing.

Carbon nanomaterials have gained significant attention in recent years, specifically in energy, biomedical, and sensing applications, driven by the desire to reduce costs and adopt more environmentally friendly practices in various industries. In electroanalytical applications, they have proven to be particularly valuable to improving electrode conductivity and active surface area [23,24]. This is attributed to their chemical inertness, wide operational potential range, versatility in different types of analyses, and outstanding electrochemical properties [25]. Carbon, as the focal element of investigation, offers a distinct advantage in forming various chemical bonds and orientations with different biological materials, owing to its diverse orbital hybridizations [26]. This has allowed for it to be studied to increase binding affinity due to Van der Waals interactions or π–π stacking. The array of carbon nanomaterial deployments includes carbon nanotubes, wires, quantum dots, graphene sheets, and graphene oxides. Each type of carbon material is adeptly applied in contexts that align with its specific structure. In biosensing development, carbon nanomaterials leverage their quantum size effects, emerging as pivotal elements. Expanding the electrode’s surface area not only enhances its active area but also facilitates greater immobilization of biological material—a crucial factor in detection. To date, GO has emerged as the frontrunner due to the electrostatic interactions possible due to oxygen inclusion. For antibody monitoring, less work has studied the electron transfer kinetics possible for conductive CNs. Consequently, this study will delve into the potential of highly conductive carbon nanomaterials including graphene quantum dots (GQDs), carbon quantum dots (CQDs), and reduced graphene oxide (rGO) for the advancement of electrochemical biosensing for SARS-CoV-2 antibodies.

This study introduces a label-free electrochemical immunosensor designed for rapid, sensitive, and specific detection of SARS-CoV-2 IgG antibodies. The sensor’s novelty lies in the incorporation of electrodeposited graphene quantum dots (GQDs), which enhance both binding efficiency and electron transfer kinetics, resulting in highly sensitive detection of Anti-SARS-CoV-2 antibodies. The use of EDC/NHS crosslinking chemistry activates the GQD-COOH functional groups, facilitating the efficient immobilization of RBD S-protein for precise detection of SARS-CoV-2 IgG antibodies while maintaining excellent current responses. The voltammetric sensor operates by disrupting the electron transfer reaction between the redox probe, Fe(CN_6_)^3−/4−^, and the active zone, a phenomenon brought about by the formation of the immunocomplex. With the global rollout of COVID-19 vaccines, it becomes imperative to develop devices capable of monitoring antibody production within the body. This step ensures that the vaccines distributed to the public remain effective, ultimately saving lives in the process.

## 2. Results and Discussion

### 2.1. Selected Characterization of Graphene Quantum Dots (GQDs)

**Figure 1:**
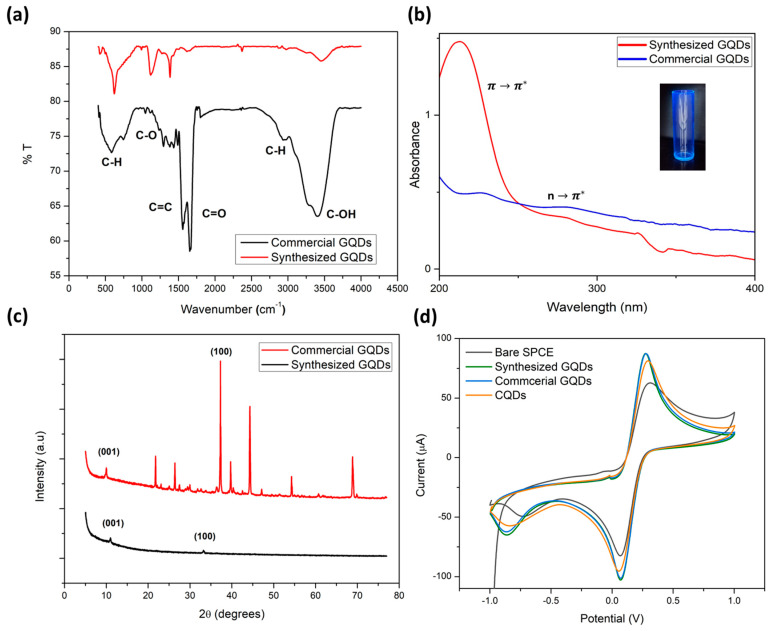
Selected characterization of commercial and synthesized graphene quantum dots (GQDs). (**a**) FTIR spectroscopy, (**b**) UV-Vis spectra, inset: blue fluorescing GQDs under UV-light excitation, (**c**) X-ray diffraction patterns, and (**d**) cyclic voltammetry in 5 mM ferricyanide in 0.1 M KCl.

FTIR analysis was conducted on synthesized and commercial GQDs to study the structural changes in the carbon-based nanomaterials, as shown in Figure 1a. The presence of carbon and oxygen containing functional groups present between 0 and 4000 cm^−1^ was confirmed in both samples. The FTIR analysis depicted in Figure 1a was carried out on both the synthesized and commercially obtained graphene quantum dots (GQDs) to examine any structural alterations in these carbon-based nanomaterials. The spectra confirmed the presence of functional groups containing carbon and oxygen within the range of 0 to 4000 cm^−1^ for both samples. Notably, the IR spectra exhibited relatively weak vibrational stretches, indicative of bond stretching vibrations associated with oxygen-containing functional groups such as -OH, COOH, -C-O, and -C-O-C-, observed at 3400, 1600, 1100, and 600 cm^−1^, respectively. Additionally, vibrations related to C-H and -C=C- within the carbon lattice were discernible at 2900 and 1300–1500 cm^−1^. These observations underscore the integration of oxygen into the carbon structure during the oxidative exfoliation process, aligning with prior research findings [27,28,29,30,31,32]. The presence of C-OH bonding at around 3400 cm^−1^ suggests the absorption of water within the lattice structures of all samples, likely due to incomplete drying. Notably, the transmittance intensities of the commercial GQDs indicated a higher degree of oxidation compared to GQDs synthesized from carbon black. This disparity may be attributed to the incorporation of oxygen within the graphite structures during the preparation process. While the elevated oxygen content in commercial GQDs may potentially reduce overall conductivity, it could enhance binding to the electrode surface through cross-linking when employed as an electrochemical biosensor.

The electronic structure of both commercially obtained and synthesized graphene quantum dots (GQDs) was examined through UV absorption spectroscopy, as illustrated in Figure 1b. The obtained spectra displayed characteristic GQD absorbance bands falling within the range from 200 to 500 nm, aligning with the observations made by Ye et al. [33]. Specifically, the UV results exhibited distinct peaks at 210 and 230 nm, attributed to π–π* transitions arising from aromatic carbon–carbon bonding resulting from C=C sp^2^ hybridization. At longer wavelengths, n–π* transitions predominated, giving rise to typical peaks at 345 nm [34]. These peaks are attributed to the sp^2^ structural domain and n–π* transition of multi-conjugate C=O [35]. Interestingly, both commercial and synthesized GQDs exhibited similar optical absorption behavior, with disparities primarily in absorbance maxima. These differences can be ascribed to reduced stacking and acidic properties. Additionally, the inset provided demonstrates the blue luminescent nature of the GQDs under UV-light, further highlighting their distinctive optical characteristics.

The structure of both the (a) synthesized graphene quantum dots (GQDs) and (b) commercial GQDs was investigated using X-ray diffraction (XRD), and the diffraction patterns are depicted in Figure 1c. This technique was employed to assess the crystallinity of the prepared and commercially obtained carbon nanostructured materials, as well as their intrinsic carbon ordering. The synthesized GQDs, derived from carbon black precursors, displayed an overall reduction in diffraction along the 002 plane, coupled with a shift to 11° resulting from diffraction in the 001 plane. This shift served as confirmation of the presence of oxygen-containing functional groups on the surface of the GQDs, corroborating the findings from the FTIR analysis. Additionally, diffraction from the 100 plane (2θ = 33°) indicated crystalline carbon stacking. The sharp peaks observed in the XRD patterns affirm the crystalline nature of the prepared GQDs, suggesting some degree of stacking prior to exfoliation. Commercial GQDs exhibited similar diffraction patterns, albeit with slight shifts in 2θ values. Notably, the commercial GQDs demonstrated enhanced crystallinity, evidenced by sharper diffraction peaks and higher intensities. The presence of additional diffraction peaks in the spectra may be attributed to the stabilizing agents employed during their synthesis [36].

Cyclic voltammetry was employed for the electrochemical characterization of the carbon nanomaterials subsequent to their electrodeposition on screen-printed carbon electrodes (SPCEs). To establish a reference, a cyclic voltammogram of the bare SPCE was initially conducted. The electroactive medium utilized was potassium ferricyanide (K_3_[Fe(CN)_6_]). Figure 1d below presents the cyclic voltammograms of the bare SPCE, GQDs-functionalized SPCE, and carbon quantum dot SPCE in a 5 mM K_3_[Fe(CN)_6_] solution using 0.1 M KCl as the electrolyte. Notably, the nanomaterials exhibited higher redox peak currents for Fe(CN)_6_^3−/4−^, indicating an enhancement in the electron transfer kinetics of the SPCE. Both the commercial and synthesized GQDs demonstrated similar performance, showcasing an approximate 50% increase in peak current. While the carbon quantum dots (CQDs) also exhibited an improvement compared to the bare SPCE, their effect was not as pronounced as that of the GQD materials. These findings underscore the significant role played by graphene quantum dots in augmenting the rate of electron transfer and the active surface area of SPCE surfaces. This enhancement holds the potential to offer heightened sensitivity in immunosensing applications.

**Figure 2:**
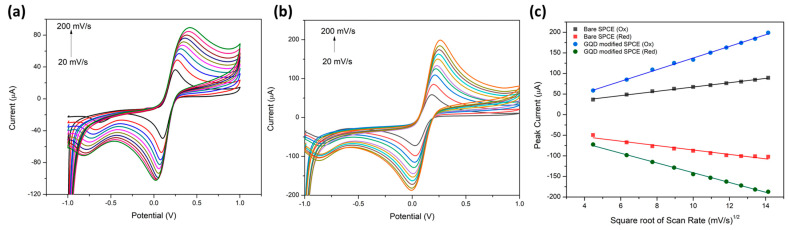
Multi-scan rate study on (**a**) bare SPCE and (**b**) GQD-modified SPCE in 5 mM ferricyanide. (**c**) Displays the linear relationship between the peak current (µA) vs. square root of the scan rate (mV/s^1/2^).

To further evaluate the electrochemical performance, experiments were conducted on both (a) the unmodified screen-printed carbon electrode (SPCE) and (b) the SPCE modified with graphene quantum dots (GQDs) using scan rates ranging from 20 to 200 mV·s^−1^. The resulting voltammograms, recorded within the voltage range from −1 to 1 V in a 5 mM ferricyanide solution, along with the corresponding current versus scan rate plots, are presented in Figure 2. An observable increase in redox peak currents was noted with the escalation of scan rate for both the GQD-modified and unmodified SPCEs (Figure 2a,b). These results indicate improved electron transfer kinetics at both electrode surfaces. Furthermore, the data obtained from the cyclic voltammograms were linearized and plotted against the square root of the scan rate, as illustrated in Figure 2c. By applying the Randles–Sevcik equation, the surface areas of the bare and GQD-modified SPCEs were determined to be 5.319 and 14.38 cm^2^, respectively. This finding demonstrates a threefold enhancement in the active surface area of the electrode, attributed to the incorporation of GQDs. This expanded surface area facilitates more effective electron transfer kinetics, showcasing a significant improvement in electrode performance.

### 2.2. High-Resolution Transmission Electron Microscopy Characterization of Carbon Nanomaterials

**Figure 3:**
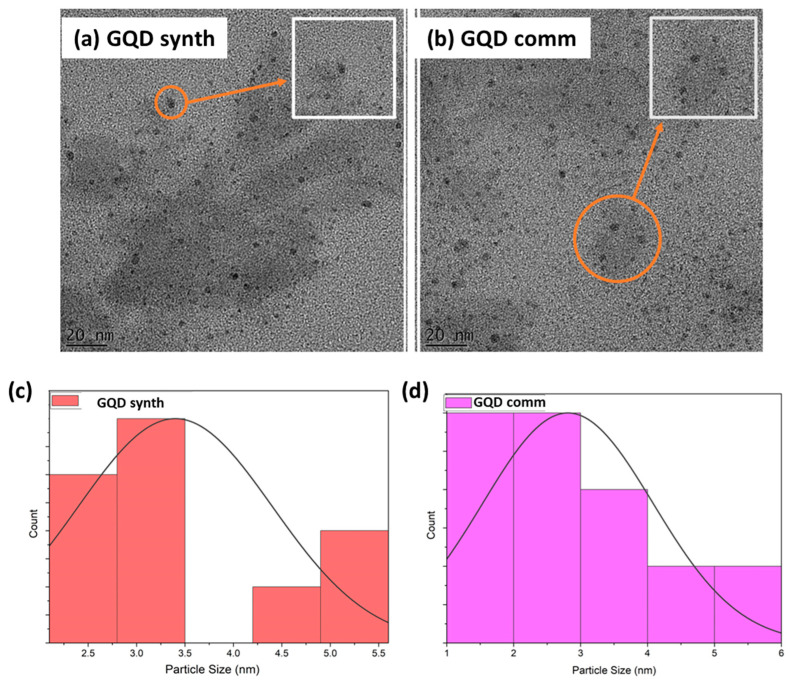
HRTEM images of (**a**) synthesized GQDs and (**b**) commercial GQDs. Size distribution curves for (**c**) synthesized GQDs and (**d**) commercial GQDs for 50 particles.

Figure 3 below provides high-resolution transmission electron microscope (HRTEM) images of both the synthesized and commercial graphene quantum dots (GQDs), accompanied by their respective size distribution plots. The observed GQDs exhibit flat, two-dimensional circular shapes, with diameters falling within the range of 3–15 nm. Notably, the crystalline nature of the GQDs is evidenced by discernible striations and lattice fringes. Moreover, the particles appear to be uniformly and densely dispersed in all samples. However, it is worth noting that the presence of excess hydrocarbons in the sample posed a challenge for imaging at higher magnifications. The findings affirm that the synthesized GQDs closely resemble the commercial GQDs in terms of size, shape, and crystalline structure. Size distribution analysis was conducted through visual imaging, demonstrating a relatively uniform size distribution in both cases. Specifically, the synthesized GQDs exhibited a size range between 2.5 and 5.5 nm, while the commercial GQDs ranged from 1 to 6 nm. The average particle sizes were determined to be approximately 3.5 nm and 3 nm for the synthesized and commercial GQDs, respectively. These results align with the spectroscopic analyses mentioned earlier.

### 2.3. Characterization of Electrochemical Carbon Nanomaterial SARS-CoV-2 Immunosensor

The electrochemical properties of the developed GQD-based immunosensor following each fabrication step were characterized through cyclic voltammetric analysis. The electrodeposition of GQDs serves as a straightforward modification of the SPCE, creating an enhanced interfacial bond between the carbon nanostructure and the electrode surface, resulting in a uniform and dense coating [37]. This step is crucial in expanding the active surface area of the SPCE, allowing for maximal immobilization of the S-protein RBD and, thus, improving binding efficiency and sensitivity. Additionally, GQDs are known to enhance electron transfer kinetics as evidenced by an increase in redox peak current, a crucial advantage in sensitive electrochemical immunosensors. It is important to note that an optimized GQD concentration should be employed to avoid potential repulsive forces between the GQDs and [Fe(CN)_6_]^3−/4−^, which could hinder electron transfer effects [38]. Following GQD modification, carboxylic groups (-COOH) on the GQDs are ready for activation via EDC/NHS chemistry. This step is pivotal, as it facilitated the immobilization of RBD S-protein onto the GQDs through the amine groups of the aforementioned antigen.

**Figure 4:**
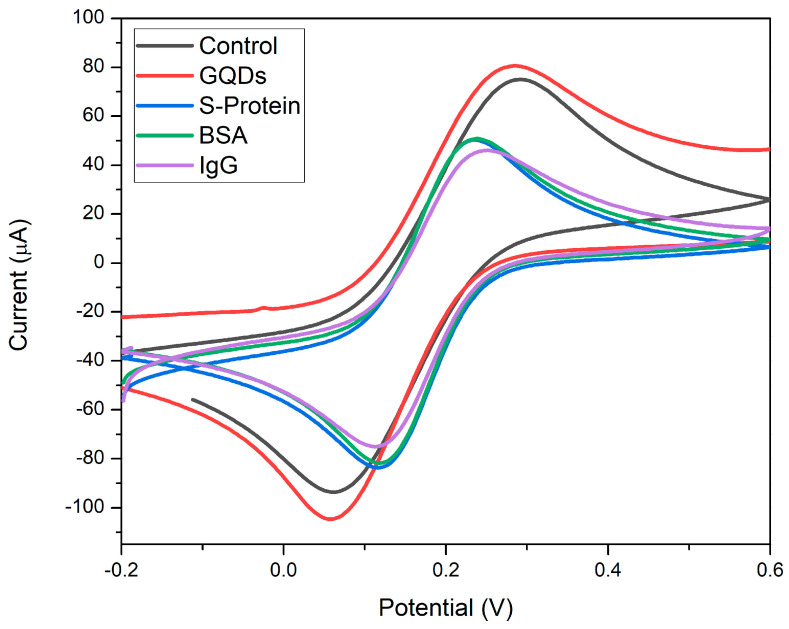
Cyclic voltammograms detailing fabrication steps of the label-free GQD-functionalized SARS-CoV-2 immunosensor in 5 mM ferricyanide between −0.2 and 0.6 V at a scan rate of 100 mV·s^−1^.

Figure 4 below illustrates the electrochemical characterization of the label-free SARS-CoV-2 immunosensor at various fabrication stages. The characteristic voltammogram for the one-electron transfer of [Fe(CN)_6_]^3−/4−^ redox couple with E_pa_ = 0.14 V and E_pc_ = 0.06 V is shown for the bare SPCE at a scan rate of 100 mV·s^−1^ between −0.2 and 0.6 V. After modification with GQDs via electrodeposition, an increase in peak currents for both oxidation and reduction peaks was observed, along with a slight narrowing in peak separation. This is attributed to the higher active surface area and improved electron transfer kinetics associated with conductive GQD modification. Upon immobilization of the RBD S-protein onto the GQD-SPCE, a significant decrease in peak height for both oxidation and reduction reactions was observed. This is a result of the non-conductive biological antigen binding, reducing the rate of electron transfer through the antigen-functionalized electrode. A 50% decrease in peak current confirms effective binding and coverage of the GQD-SPCE surface via EDC/NHS crosslinking. Following the addition of BSA, minimal change was observed in the voltammogram, indicating consistent electron transfer reactions. Bovine serum albumin (BSA) served as a blocking agent to reduce non-specific binding at the electrode surface. Incubation of 10 ng·mL^−1^ SARS-CoV-2 IgG antibody resulted in the formation of an immunocomplex with the immobilized RBD S-protein, inhibiting electron transfer reactions. This led to a decrease in peak height at both oxidation and reduction peaks, centered at E_pa_ = 0.22 V and E_pc_ = 0.12 V, respectively. In conclusion, the decrease in peak heights in both oxidation and reduction reactions due to IgG antibody binding indicates a decline in electron transfer kinetics at the electrode surface, attributed to the formation of the immunocomplex of SARS-CoV-2 S-protein and IgG antibody. These results affirm the successful development of the novel immunosensor and suggest its potential application for the detection of SARS-CoV-2-specific antibodies in test samples.

### 2.4. Assay Optimization

The sensitivity of the developed sensor was controlled by the efficient binding of bioreceptor at the SPCE surface. The GQD concentration, EDC/NHS ratio, concentration, and incubation time as well as RBD S-protein antigen and 4% BSA used to block non-specific sites on the immunosensing platform were optimized.

**Figure 5:**
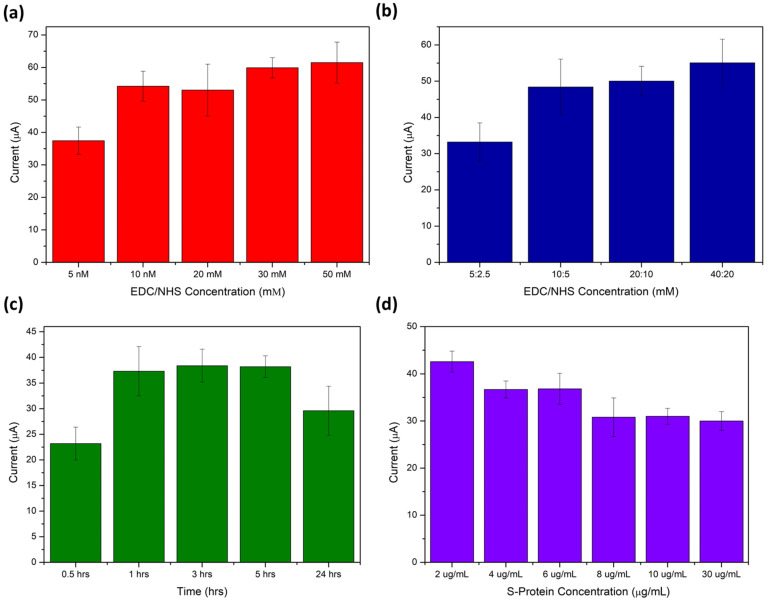
Optimization studies of (**a**) EDC/NHS concentration, (**b**) EDC/NHS ratios, (**c**) anti-SARS-CoV-2 S-RBD protein incubation time, and (**d**) concentration (*n* = 3).

The concentration of the EDC/NHS crosslinker is vital to the achieving maximum binding efficiency of the bioreceptor. EDC/NHS chemistry plays a pivotal role in this process. EDC was utilized to activate the -COOH functional groups present on the GQDs, facilitating immobilization via the amine groups of the RBD S-protein antigen onto the electrode’s surface through NHS crosslinking [39]. Figure 5 illustrates the optimization of the EDC/NHS concentration for immobilizing the RBD S-protein antigen on the SPCE surface. A 1:1 EDC/NHS ratio was studied at concentrations of 5, 10, 20, 30, and 50 mM. It was observed that as the concentration increased within the studied range, the oxidation peak current of 5 mM ferricyanide at a GQD-modified SPCE also increased. The EDC/NHS crosslinking altered the surface charge of the electrode, allowing for greater accumulation of the ferri/ferrocyanide anions at the electrode surface. A concentration of 10 mM of EDC/NHS showed sufficient activation of the GQD carboxylic acid groups and was chosen for further investigation. Figure 5b demonstrates an increasing trend in current peak height with escalating concentrations of EDC/NHS in a 2:1 ratio. Higher concentrations showed improved activation of the GQD surface for crosslinking; contrastingly, altering the EDC to NHS ratio had minimal influence on surface activation. A 10:5 mM EDC/NHS concentration was selected for further studies to ensure complete activation of the electrode surface. These findings indicate that lower EDC/NHS concentrations are sufficient in inducing the necessary surface changes for immunosensor fabrication. Furthermore, an excess of EDC can potentially reduce the sensitivity of the immunosensor, as EDC has a tendency to form by-products with the antibody/antigen [40].

The passive adsorption of spike protein receptor-binding domains (RBDs) onto the GQD-modified electrode surface offers a straightforward method for electrode functionalization. During this process, various non-covalent interactions are at play, governed by the EDC/NHS crosslinker. Incubation time plays a crucial role in determining the distribution of RBD proteins across the GQD-SPCE surface.

In Figure 5c, a time-dependent study was conducted using a 5 µg/mL RBD S-protein sample immobilized on a 0.5 mg/mL GQD-SPCE surface. The incubation times were varied between 0.5, 1, 3, and 24 h, and the resulting [Fe(CN)_6_]^3−/4−^ oxidation peak current was observed. An increase in peak current was observed up to an incubation time of 1 h. Beyond this point, saturation of RBD proteins at the electrode surface was evident, with little-to-no change in peak current. The results indicate that the maximum current response was achieved at an incubation time of 3 h. Incubating RBDs for up to 24 h resulted in the complete blocking of the electrode surface with non-conductive protein, significantly impeding electron transfer at the electrode surface. Therefore, an incubation time of 1 h was selected for RBD S-protein immobilization in the immunosensor. This incubation period demonstrated significant RBD-functionalization even at relatively short times.

Following this experiment, a concentration study of the RBD S-protein was conducted, as shown in Figure 5d. Concentrations of 2, 4, 6, 8, 10, and 30 µg/mL were used. At higher concentrations of the antigen immobilized on the GQD-SPCE surface, there was a tendency to reduce the electron transfer of [Fe(CN)_6_]^3−/4−^, leading to a decrease in oxidation peak current. Saturation of the GQD-SPCE surface with S-protein RBD antigens occurred at concentrations greater than 8 µg/mL. Beyond this point, no significant change in peak current was observed, indicating complete coverage of the electrode surface with RBD bioreceptor. Consequently, the optimized concentration for the immobilization of RBD S-protein is 8 µg/mL, and this was utilized in the fabrication of the immunosensor.

### 2.5. Carbon Nanomaterial-Modified SARS-CoV-2 Immunosensor Label-Free Detection of SARS-CoV-2 Antibodies

**Figure 6:**
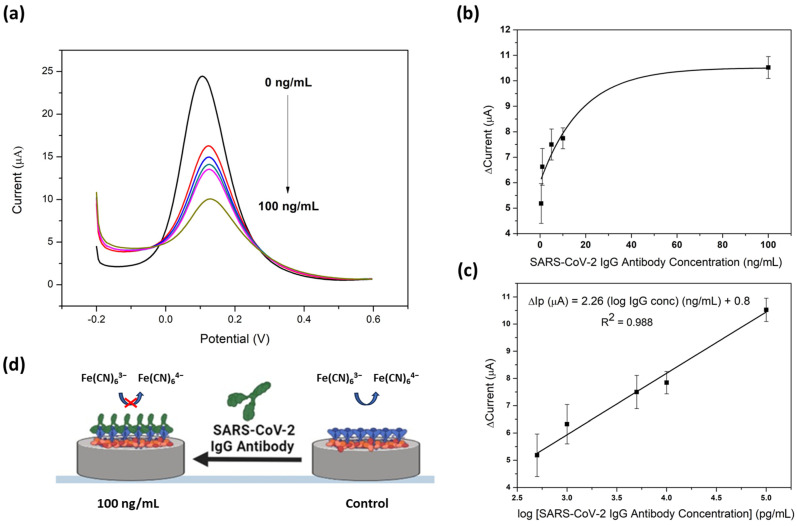
(**a**) DPV detection of SARS-CoV-2 IgG antibodies via a RBD S-protein immune complex immobilized onto a GQD-SPCE in 5 mM ferricyanide between 0 and 100 ng/mL, (**b**) corresponding calibration curve between Δ current and concentration of SARS-CoV-2 IgG, and (**c**) Δ current and log of concentration of SARS-CoV-2 IgG (*n* = 3). (**d**) Schematic of the novel GQD-functionalized immunosensor.

The GQD-functionalized immunosensor was applied to the detection of SARS-CoV-2 IgG antibodies in test samples. The detection was conducted using potassium ferricyanide (5 mM) as a redox probe, where the change in current was monitored as the concentration of the SARS-CoV-2 antibodies increased between 0 and 100 ng/mL. Figure 6a shows the voltammograms recorded between −0.2 and 0.6 V. As the concentration of the antibody is increased, a decrease in oxidation peak current was observed. This was due to the disrupting of the potassium ferricyanide oxidation reaction that occurs when the immunocomplex forms between the SARS-CoV-2 RBD S-protein antigen and the SARS-CoV-2 IgG antibody at the GQD surface.

Figure 6b displays the corresponding average calibration curves for three replications indicating the relationship between the change in current and the SARS-CoV-2 IgG antibody concentration. A logarithmic relationship between ferricyanide oxidation current and concentration was observed across the 0.5–100 ng/mL range. The calibration curve was further linearized by plotting the logarithmic concentration (Figure 6c). The immunosensor developed displayed sensitive detection of SARS-CoV-2 IgG antibodies with a sensitivity of 2.126 µA/pg·mL^−1^. The change in current was due to the amount of binding that occurs between the RBD S-protein and IgG antibody. The IgG immunocomplex formed reduced the amount of redox probe which could access the electrode surface. It is seen that the change in current was proportional to the logarithmic concentration of the SARS-CoV-2 antibody, resulting in a linear plot. The change in current increased with the logarithmic concentration. For this COVID-19 immunosensor, the dynamic linear range was found to be between 0.5 and 100 ng/mL. This resulted in an R^2^ value of 0.9599, indicating good regression for the biosensor. Using the equation (3SD_blank_/slope), the LOD was determined to be 2.028 ng/mL. A schematic of the developed GQD-modified immunosensor is shown in Figure 6d.

Table 1 summarizes the recorded recovery (%) of the GCQ-SPCE for the detection of 5 ng/mL SARS-CoV-2 IgG antibodies. The sensor showed excellent accuracy in detecting SARS-CoV-2 IgG antibodies with a recovery % of 99.2 ± 7.8% for the three replications.

### 2.6. Reproducibility Study for the Detection of SARS-CoV-2-Specific IgG Antibody via GQD-Modified SARS-CoV-2 Immunosensor

**Figure 7:**
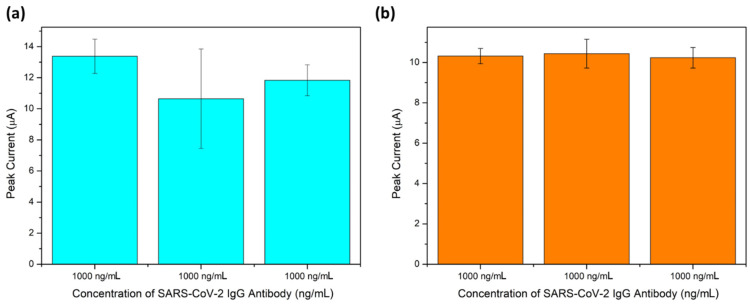
Bar graph displaying the reproducibility of the developed GQD-modified SARS-CoV-2 immunosensor in 5 mM ferricyanide (*n* = 3) for incubations on (**a**) different and (**b**) the same SPCEs (*n* = 3).

Figure 7 illustrates the reproducibility of the SARS-CoV-2 IgG antibody detection process for a concentration of 1000 ng/mL, comparing two different conditions: (a) incubation on different GQD-SPCEs; (b) incubation on the same GQD-SPCE for three replications (*n* = 3). The standard deviation of the SARS-CoV-2 immunosensor was determined to be 1.368 for incubation on different SPCEs, and 0.1007 for incubation on the same SPCE. The process of immobilizing the antibody on the same electrode was achieved by first cleaning the SPCE with bovine serum albumin (BSA), followed by re-immobilizing the analyte. This method significantly improved the reproducibility of the immunosensor. The relative standard deviation percentages (RSD%) were found to be 11.45 and 0.97 % for the different and same GQD-SPCEs, respectively. Based on these findings, it is concluded that incubating the antibody on the same electrode leads to significantly better reproducibility. Therefore, this method can be adopted for the fabrication of the SARS-CoV-2 immunosensor in future experiments.

### 2.7. SARS-CoV-2 Antibody Detection Immunosensor Using Other Carbon Nanomaterials

**Figure 8:**
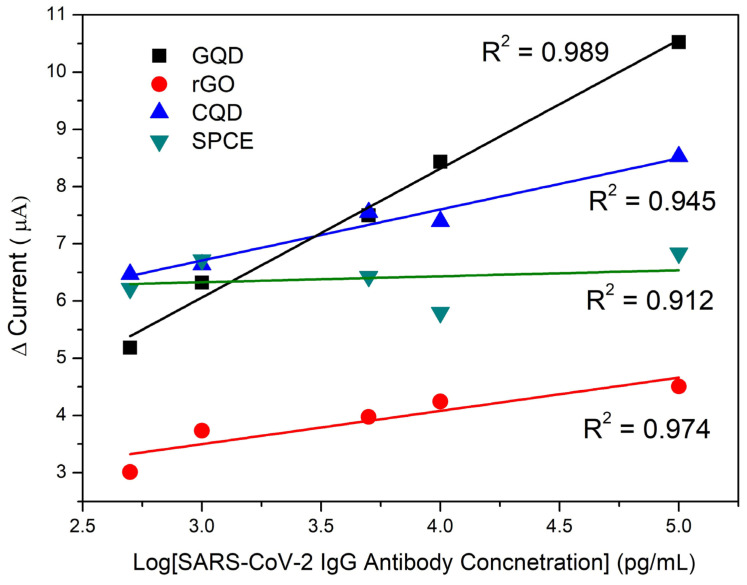
Calibration curves recorded for low-oxygen content CN-modified SARS-CoV-2 immunosensor for 2.5–5 pg/mL Anti-SARS-CoV-2 IgG Antibodies at GQD, rGO, CQD, and SPCEs.

The developed GQD-immunosensor was compared to other highly conductive and low-oxygen-content carbon nanomaterials (electrochemically reduced graphene oxide and carbon quantum dots). The sensitivity of the carbon nanostructured immunosensors was compared to the GQD-SPCE immunosensor for the detection of SARS-CoV-2-specific IgG antibodies under the optimized designed conditions. The recorded calibration plots are shown in Figure 8 for 2.5–5 pg/mL SARS-CoV-2-specific IgG antibodies.

The unmodified immunosensor demonstrated low sensitivity toward the IgG antibody detection. The low sensitivity is attributed to the absence of suitable functionalities at the electrode surface required for suitable S-protein RBD binding as described in previous sections. As a result, irreproducibility of the developed sensor and low linear correlation were found. The rGO platform demonstrated very little binding of the immunocomplex due to the absence of oxygen-containing moieties and was found to be unsuitable for detection even though high conductivity was expected. CQDs showed low detection sensitivity along with the synthesized GQDs in this study. The lower sensitivity and current responses may have arose from the spherical nature of the carbon nanomaterials compared to the 2D sheets seen in the other nanomaterials under investigation. Improved linearity, however, was observed. The immunosensor developed from GQDs, however, showed the highest current responses of all platforms studied. The high oxidation peak currents demonstrate that the GQDs are highly conductive and offer the highest active surface area and binding efficiency.

Table 2 summarizes and compares the statistical values resulting from the performance of the respective SARS-CoV-2 immunosensors fabricated with various carbon nanomaterials. Comparing the slopes of the investigated immunosensors, it is seen that the immunosensors of the carbon nanomaterial-functionalized platforms offer comparable or improved results compared to reported studies. The bulk of work shown relied on patient sampling instead of sensor development. The bare SPCE immunosensor performed the worst due to instability and fluctuations in binding efficiency resulting in changing SPCE oxygen content. The low regression suggests that CN-functionalization is required for improved performance. The GQD-based immunosensor performed the best with the highest sensitivity of 2.12 µA/pg·mL^−1^. The immunosensor fabricated with GQDs further offers good stability and linear regression in the concentration range studied. The electrochemically reduced graphene oxide further suggested good results due to stable rGO films at the SPCE surface and edge-like oxygen moieties remaining following reduction, offering good binding of the S-protein RBD protein bioreceptors. Moreover, LODs ranged from 2.03 to 41.1 ng/mL for all CN-functionalized sensors with GQDs, offering the best results. While many works report the detection of SARS-CoV-2 antigens, to date, only this work reports the anti-SARS-CoV-2 antibody detection centers around immunosensing due to the selective antibody-antigen interactions. No other biosensors have been reported for antibody detection.

### 2.8. GQD-Modified SARS-CoV-2 Immunosensor Label-Free Detection of SARS-CoV-2 Antibodies in Real Sample

**Figure 9:**
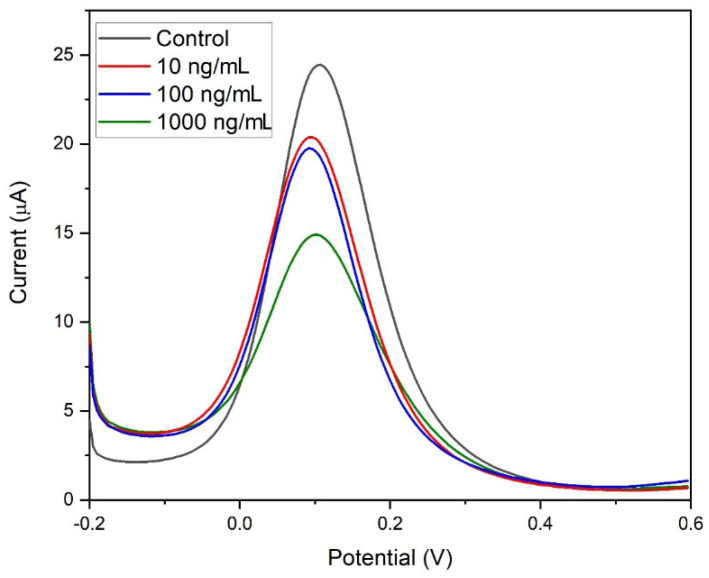
DPV detection of SARS-CoV-2 IgG antibodies via a RBD S-protein immunocomplex immobilized onto a GQD-SPCE immunosensor in human serum at a 1:10 ratio in 5 mM ferricyanide.

The developed label-free GQD-modified SPCE SARS-CoV-2 immunosensor was tested in a synthetic real sample using human serum mixed in a 1:10 ratio with PBS to enhance conductivity. This allowed for the electrochemical detection of SARS-CoV-2 IgG antibodies. Figure 9 displays the DPV detection of SARS-CoV-2 IgG antibodies in human serum. The experiment demonstrates the effectiveness of the immunosensor with the GQD modification and a 5 mM ferricyanide redox probe. The successful detection of specific IgG antibodies in human serum was indicated by a decrease in current with increasing concentration. This suggests a successful binding between the IgG antibody and the immobilized RBD S-protein, forming an immunocomplex. The formation of this immunocomplex reduces the exposure of the electrode surface to the human serum, affecting the electron transfer kinetics. This breakthrough in successful detection in human serum opens a new platform for SARS-CoV-2 immunosensors, which not only detect the virus but also offer potential for monitoring the efficacy of SARS-CoV-2 vaccinations.

## 3. Materials and Instrumentation

### 3.1. Materials and Chemicals

Carbon black (Sigma-Aldrich, St. Louis, MI, USA), 98% sulphuric acid (Sigma-Aldrich), nitric acid (Sigma Aldrich), ultrapure water, NaOH (Sigma-Aldrich), L-histidine (Sigma-Aldrich), Citric Acid (Sigma-Aldrich), potassium ferricyanide, phosphate buffer solution, biological phosphate buffer solution (Sigma-Aldrich), 1-ethyl-3-(-3-dimethylaminopropyl) carbodiimide hydrochloride (Sigma-Aldrich), N-Hydroxysuccinimide (Sigma-Aldrich), SARS-CoV-2 spike protein (Sino-Biological, Beijing, China), bovine serum albumin (Sigma-Aldrich), SARS-CoV-2 immunoglobin g (Sino-Biological), immunoglobin m (Sino-Biological) blue luminescent graphene quantum dots (Sigma-Aldrich), blue luminescent graphene quantum dots (Advanced Chemicals Supplier, Pasadena, CA, USA), human serum (Sino-Biological, Beijing, China).

### 3.2. Instrumentation

Bransoic, Branson 2800-MH Sonicator (Sigma-Aldrich, Darmstadt, Germany), hot plate (Heidolph MR Hi-Standard, Lasec, Cape Town, South Africa), rotor evaporator- Büchi Rotavapor R-114 (Sigma-Aldrich, Darmstadt, Germany), microwave (Anton Paar Microwave Reaction System SOLV Multiwave PRO, Anton Paar Australia Pty. Ltd., North Ryde, New South Wales), UV-VIS (Varian Cary 300, Agilent, Santa Clara, CA, USA), PL (Horiba Scientific Nanolog, Gauteng, South Africa), FTIR (PerkinElmer Spectrum Two, PerkinElmer Co., Ltd., Midrand, South Africa), Malvern Zeta Sizer (Malvern Instruments Ltd., Great Malvern, UK), Malvern zeta potential folded capillary cell (Malvern Instruments Ltd.), Potentiostat (Metrohm AutoLab PGSTAT 101, Utrecht, The Netherlands).

### 3.3. Synthesis of Graphene Quantum Dots

This work reports a modified inexpensive facile one-step wet-chemistry technique for the synthesis of GQD employed by Ye et al. [33]. The difference in the synthesis is found in the use of carbon black as the source of carbon rather than coal. Following the simple procedure, the resulting synthesized GQDs were cooled to room temperature where they were poured into ice and the pH was adjusted to 7. The solution of GQDs was then washed with deionized water and placed into 3000, 6000, and 12,000 Da dialysis bags for a week. The collected solution was then placed in a vacuum oven to dry overnight at 80 °C, resulting in dry GQDs.

### 3.4. Synthesis of Carbon Quantum Dots

This work reports a solvo-thermal synthesis technique for the synthesis of CQD by Quin et al. [41]. Following a simple procedure where citric acid was the carbon source, CQDs were synthesized using a microwave at 800 watts. The solution turned brown and was left to cool to room temperature. Following this, the solution was centrifuged at 12,000 rpm for 30 min, where the pellet was discarded and supernatant was kept. Upon the collection of the supernatant, dialysis was performed on the supernatant for a week in two different dialysis bag sizes: 3000 and 6000 Da.

### 3.5. Fabrication of GQD-Functionalized Screen-Printed-Carbon Electrode Immunosensor

**Figure 10:**
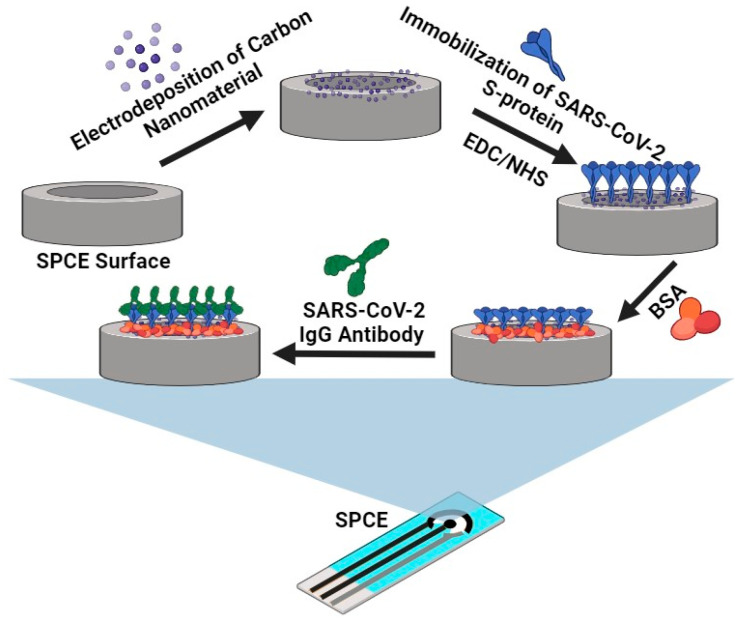
Schematic diagram displaying the fabrication procedure for the GQD-modified SARS-CoV-2 SPCE immunosensor.

A 100 µL solution of graphene quantum dots (GQDs) with a concentration of 0.5 mg/mL, dispersed in 0.1 M phosphate-buffered solution (PBS) at pH 7.4, was electrodeposited onto the surface of a screen-printed carbon electrode (SPCE) using a fixed potential reduction of −0.3 V for a duration of 5 min. Subsequently, the GQD-coated SPCE was annealed in an oven at 60 °C for 15 min to enhance adhesion and remove any residual moisture. After cooling to room temperature, a mixture of 20 µL of EDC/NHS (in a 2:1 ratio, equating to 10:5 mM) was carefully applied to the modified SPCE surface. This was left at room temperature for one hour to activate the -COOH groups on the GQD-coated SPCE. Following activation, the excess EDC/NHS was rinsed off the electrode, after which 20 µL of SARS-CoV-2-specific RBD S-protein antigens (at a concentration of 8 µg/mL) was immobilized onto the activated GQD-coated SPCE surface. This mixture was then incubated at 4 °C for a period of one hour. To block any remaining binding sites on the S-protein, 20 µL of 4% BSA was carefully applied to the modified electrode surface and allowed to incubate for 30 min at 4 °C. The entire procedure is illustrated in Figure 10 below.

### 3.6. Electrochemical Detection of SARS-CoV-2 IgG and IgM Antibodies Using Carbon Nanomaterial-Modified SPCE’s

The IgG/IgM antibodies were dropped onto the activated GQD-SPCE. The complete sensor was then incubated for 15 min at 4 °C. Upon the completion of binding between the IgG/IgM antibodies to form an immunocomplex, the immunosensor was rinsed gently with biological PBS to remove all unbound antibodies. The immunosensor was then subjected to differential-pulse voltammetry (DPV) for the detection of IgG /IgM SARS-CoV-2 antibodies, using 5 mM solution of [Fe(CN)_6_]^3−/4−^ in 0.1 M KCl as the redox probe on the reaction zone. The DPV technique monitors the change in current produced due to antibody binding compared to the antibody-free control experiment (bare). The immunocomplex formed hinders the flow of electrons, which in turn hinders the reactions of the redox probe, showing lower currents on the DPV voltammogram.

## 4. Conclusions

This study presents an electrochemical immunosensor employing GQD-modified SPCEs for the highly sensitive label-free detection of SARS-CoV-2 IgG antibodies. The sensing mechanism hinges on the perturbation of redox reactions and electron transfer kinetics of the Fe(CN)₆^3−^/^4−^ redox probe, driven by the formation of an immunecomplex between the specific RBD S-protein antigen and IgG antibody. GQDs played a pivotal role in augmenting detection sensitivity and enlarging the active surface area of the working electrode, leading to enhanced binding efficiency of the immunocomplex. Notably, individual SPCEs exhibited markedly improved reproducibility compared to disposable single concentration detection. The achieved low limit of detection (LOD) of 2028 ng/mL underscores the high sensitivity of the sensor, on par with similar immunosensors developed. Furthermore, the developed BSA/RBD/GQD-SPCE platform demonstrated proof of concept detection for human IgM antibodies, exhibiting minimal interference in the presence of other antibodies. The GQD immunosensor demonstrated superior detection capabilities compared to other low-oxygen content carbon nanostructured platforms studied. The findings suggest that although electrode conductivity may be improved by relying on carbon–carbon conjugated carbon nanostructures, binding affinity plays the crucial role in the performance of the electrochemical immunosensor. For this purpose, greater oxygen may be required for improved sensing efficiency at the expense of improved electron transport. This point of care (POC) device, leveraging GQDs, furnishes a versatile platform adaptable to detect various infectious diseases, constituting a substantial advancement in global healthcare. Moreover, the novel immunosensor will be deployed to monitor IgG antibody production in SARS-CoV-2 vaccinated individuals, furnishing crucial insights into vaccine effectiveness and durability in an African context. Crucially, the developed sensor demonstrates its efficacy in detecting real samples, vital for practical implementation in resource-constrained settings. In essence, this work not only advances SARS-CoV-2 detection capabilities but also showcases the broader potential of this technology in healthcare diagnostics and further elucidates the role of carbon nanostructures in electrochemical immunosensing.

## Figures and Tables

**Table 1:** Recovery percentages for the detection of SARS-CoV-2 IgG antibodies at the GQD-SPCE.

Replication	Spiked (ng/mL)	Detected (ng/mL)	RSD%
1	5	5.22	104.4
2	5	5.16	103.2
3	5	4.51	90.2

**Table 2:** Comparison of SARS-CoV-2 immunosensors using various carbon nanomaterials for the fabrication of the SPCE.

Platform	Target Antibody	Slope	R^2^	LOD (ng/mL)	Reference
Pept/AuNP-SPCE	Anti-S	1.059	0.984	75	[16]
prGOx-Au-coated multiplex sensor chip	Anti-SARS-CoV-2	N/A	N/A	N/A	[17]
Streptavidin/biotin SPCE	ALP-anti human IgG and IgM	N/A	N/A	1.64	[18]
Au-TNP wells	anti human IgG and IgM	N/A	N/A	89 aM	[19]
Multiplexed sensor chip	anti human IgG	N/A	N/A	2.3 copies/µL	[20]
Interdigitated Au	Anti-SARS-CoV-2 monoclonal	N/A	0.9	1000	[21]
GO-paper-based carbone electrode	S-protein IgG and IgM	6.1137	0.995	0.14	[22]
SPCE	S-protein IgG	0.1046	0.9129	41.140	This work
GQD-SPCE	S-protein IgG	2.1216	0.9885	2.028	This work
CQD-SPCE	S-protein IgG	0.9677	0.9429	4.447	This work
rGO-SPCE	S-protein IgG	0.5807	0.9740	7.410	This work

Alkaline Phosphate label (ALP), Pentaamine-modified graphene nanoflakes (prGOx), Peptide-Gold Nanoparticle (Pept/AuNP), Gold triangular nanoprisms (Au-TNP), Screen Printed Carbon Electrode (SPCE), Graphene Quantum Dot (GQD), Carbon Quantum Dot (CQD), Reduced Graphene Oxide (rGO). N/A—Not applicable.

## Data Availability

All research data can be found within this publication.
